# Gender differences in hypertension awareness, antihypertensive use and blood pressure control in Bangladeshi adults: findings from a national cross-sectional survey

**DOI:** 10.1186/s41043-017-0101-5

**Published:** 2017-05-25

**Authors:** Muntasirur Rahman, Gail Williams, Abdullah Al Mamun

**Affiliations:** School of Public Health, The University of Queensland, Herston Road, Herston, QLD 4006 Australia

**Keywords:** Blood pressure, Hypertension, Awareness, Gender, Public health

## Abstract

**Background:**

Bangladesh is facing an epidemiological transition with a growing burden of non-communicable diseases. Traditionally, hypertension and associated complications in women receive less recognition, and there is a dearth of related publications. The study aims to explore gender differences in high blood pressure awareness and antihypertensive use in Bangladeshi adults at the community level. Another objective is to identify factors associated with uncontrolled hypertension among antihypertensive users.

**Methods:**

Data from the Bangladesh Demographic and Health Survey (BDHS 2011) was analysed. From a nationally representative sample of 3870 males and 3955 females, aged ≥35 years, blood pressure and related information were collected following WHO guidelines. Logistic regression models were used to estimate adjusted odds ratio (AOR) for factors affecting blood pressure awareness, antihypertensive use and uncontrolled hypertension among males and females taking antihypertensive medications. All analyses were weighted according to the complex survey design.

**Results:**

Women were more likely to have their blood pressure measured (76% vs. males 71%, *p* < 0.001) and to be ‘aware’ about their own high BP (55% vs. males 43%, *p* < 0.001). No gender difference was observed in antihypertensive medication use among those who were aware of their own high BP (females 67%, males 65%, *p* = 0.39). Non-working females were less likely to use antihypertensive (67% vs. non-working males 77%, *p* < 0.05). Poor women were worse off compared with poor males in antihypertensive medication use. One-in-three antihypertensive medication users had stage 2 hypertension (SBP ≥160/DBP ≥100 mmHg). Female sex, older age, increased wealth, higher BMI and certain geographical regions were associated with poor blood pressure control among antihypertensive medication users.

**Conclusions:**

BP check-ups and hypertension awareness were higher among women than men but did not translate into better antihypertensive medication practice. Gender disadvantage and inequity were observed in antihypertensive medication use. Our findings reiterate the importance of sex-disaggregated analysis and reporting. Policy makers should explore the uncontrolled hypertension burden and geographical variations in Bangladesh.

## Background

Raised blood pressure (BP) is the largest contributor to the global burden of disease and mortality, leading to approximately 9.4 million deaths annually [1]. Hypertension (≥140/90 mmHg) is present in more than a quarter of the adult world population [2]. Prehypertension (≥120–139/80–89 mmHg) has also been associated with the development of hypertension and diabetes mellitus and increased risk of myocardial infarction, stroke and cardiovascular diseases [3]. About 54% of stroke, 47% of ischaemic heart disease and 25% of other cardiovascular diseases (CVDs) worldwide were attributable to high BP [4]. Hypertension coexists with chronic kidney disease in 67–92% of cases [5].

Undiagnosed hypertension and uncontrolled hypertension despite antihypertensive treatment are global public health challenges. Most recommendations suggest lowering the systolic BP (SBP) and diastolic BP (DBP) to values within the 130–139 and 80–85 mmHg range, in all hypertensive patients, to prevent associated complications and mortality [6]. Awareness, treatment and control of hypertension in lower income countries (LICs) are low, 41, 32 and 40%, respectively [7]. A systematic review found hypertension awareness, treatment and control in India ranged from 12 to 54%, 8 to 47% and 7.5 to 25%, respectively [8]. In a rural area of Bangladesh, hypertension prevalence was as high as 40% but hypertension awareness was only 18% [9].

Bangladesh is one of the few developing countries in the world on track to meet Millennium Development Goal (MDG) 4 and 5 that is reduction in under-five mortality and maternal mortality [10]. This country of approximately 160 million people is going through an epidemiological transition with large increases in chronic diseases [11]. Over a 20-year period (1986 to 2006), CVDs increased by 30-fold among males and by 46-fold among females [12]. Among those 15 years or older in a rural area of Bangladesh, non-communicable diseases (NCDs) were responsible for about 51% of the reported deaths, followed by communicable diseases (23%); stroke and cardiac diseases were the leading causes [13, 14]. However, Bangladesh is still to roll out routine surveillance of chronic diseases, and population level data on awareness, prevention and management of hypertension is very limited.

Hypertension and its complications are usually perceived as men’s disease, yet contrary to the popular belief, hypertension and cardiovascular events are higher in women, especially in post-menopause. Women were twice more likely than men to have uncontrolled hypertension in older age [15] and experience higher CVD outcomes in later life, mostly due to longevity and hormonal changes [16]. The effects of elevated BP, cholesterol and body weight on CVD outcomes are mostly similar between women and men [17]. However, often women do not receive optimal management for high BP and experience poor outcomes, compared with men [18]. Very few physicians are even aware of increased future hypertension, diabetes or CVD risks in women with pre-eclampsia [19]. Women representation and sex-specific findings are disproportionately low in CVD related researches [20].

In this paper, we have explored whether there is any difference in hypertension awareness, prescribed antihypertensive medication use and BP control among Bangladeshi men and women, which so far has been little explored. Such findings from a nationally representative sample of adults are expected to help policy makers and healthcare providers in recognizing any existing inequity and unmet needs in hypertension management in the country. The findings are also anticipated to raise hypertension awareness and inform the design of appropriate gender-specific public health interventions in Bangladesh and other developing countries.

## Methods

### Data source

For this study, we did secondary analysis of data from the Bangladesh Demographic and Health Survey 2011 (6th BDHS). The study population comprised a nationally representative sample of women and men aged 35 years and older. Participants were identified using multistage sampling from a geographically clustered, probability-based sample of households. Data collection was carried out between July and December, 2011. Participation rate was high, about 86% for men (*N* = 4524) and 92% for women (*N* = 4311). All subjects provided informed consent. The detailed methodology and data collection procedure of BDHS 2011 have been described previously [21].

### Outcome

i) Measurement and categorization of BP
  Blood pressure was measured using LifeSource® UA-767 Plus automatic blood pressure monitor, as recommended by the World Health Organization (WHO) [21]. Small, medium or large cuffs were used according to arm circumferences of the respondents. Three measurements were taken by trained health technicians, at seating position, at approximately 10-min intervals. The average of the second and third measurements was used to record SBP and DBP.
  BP data was categorised using the American Heart Association guidelines for cut-off points (22). A SBP less than 120 mmHg and DBP less than 80 mmHg were considered normal. Prehypertension was categorised as a SBP value of 120–139 mmHg or a DBP value of 80–89 mmHg. A SBP of 140 mmHg or higher or a DBP of 90 mmHg or higher was categorised as hypertension. Current users of antihypertensive medication were also categorised as having hypertension.
  High BP values were further classified into stage 1 hypertension, defined as a SBP 140–159 mmHg, or a DBP 90–99 mmHg and stage 2 hypertension, defined as SBP of 160 mmHg or higher or DBP of 100 mmHg or higher. According to most guidelines, stage 2 hypertension is a serious form of high blood pressure, which requires immediate treatment. For stage 1 hypertension, guidelines mostly recommend lifestyle change (without medication) to avoid progression to stage 2 hypertension and future CVD complications [21].
ii) Awareness, treatment and control of hypertension
  All respondents were asked whether they (i) ever checked (before the survey) their BP, (ii) ever told by a doctor or nurse that they had high BP and (ii) were taking any prescribed medication to lower their BP.
*Hypertension awareness*: Self-reported knowledge about own high blood pressure, from a physician or nurse, among individuals who were identified with hypertension (SBP ≥140 mmHg or DBP ≥90 mmHg) during the survey or reported taking prescribed BP lowering medications.
*Antihypertensive treatment*: Self-reported use of prescribed BP-lowering medications. The study did not collect detailed information about medication such as the name, dose or duration of treatment.
*Control of hypertension*: A person treated with prescribed BP-lowering medication and having an SBP value of less than 140 mmHg and a DBP value of less than 90 mmHg.

### Measurement of covariates

The following DHS variables were considered as potential determinants for BP measurement (awareness), antihypertensive use and uncontrolled BP among the antihypertensive medication users

i) Demographic variables
  Age at the time of interview –35 to 55 years (younger) and 56 years or more (older) and sex (male, female) of the respondents.
ii) Regional variables
  Area of residence (rural and urban) and region of residence. Seven administrative regions were Dhaka (central), Chittagong (south-east), Sylhet (east), Barisal (south), Khulna (west), Rajshahi (mid-west) and Rangpur (north-west).
iii) Socio-economic variables
  Respondent’s highest educational attainment, wealth status, current working status and current marital status.
  Wealth quintile is an indicator of within country relative wealth according to household assets. For wealth status, respondents in the lowest two quintiles were categorised together (poorest or poor); similarly, upper two quintiles were categorised together (richer or richest). The wealth index was constructed using household asset data via principal components analysis. Information on house materials, sources of drinking water, sanitation facilities, use of soap and water for hand washing, availability of electricity, housing amenities, possession of household durable goods and home and land ownership were used in the construction of wealth quintiles [21].
iv) Nutritional variables
  Height (in metres) and weight (in kilograms) of the respondents were measured using a standard protocol [21]. Body mass index (kg/m^2^) was calculated dividing weight by height squared. BMI values were categorised as thin (<18.5), normal (≥18.5 but <25), overweight/obese (≥25), using WHO classification [22]. We have combined overweight (BMI 25 to <30) and obesity (BMI ≥30) for analytical purposes as the prevalence of obesity was low.

### Statistical analysis

We initially provided descriptive information of the selected variables including characteristics of the study population, separately for males and females. Chi-square tests were used to assess the proportional differences between males and females. Then, distribution of the dependent variables (outcome of interests) such as prior BP measurement, knowledge about own hypertension, antihypertensive medication use and BP control among antihypertensive medication users were assessed across the selected categorical variables, again separately for males and females.

We used multiple logistic regression models to calculate adjusted odds ratio (AOR) with 95% confidence interval (CI) for factors affecting prior BP measurement, hypertension awareness, antihypertensive use and control of hypertension, separately for males and females. We considered all potential determinants in the same model to estimate the AOR. All analyses incorporated the complex sampling design of BDHS 2011 survey. A *p* value of ≤0.05 was considered as statistically significant.

Statistical analysis was performed using Stata version 12 (Stata Corp., Texas) for Windows.

## Results

The mean age of the respondents was 51.4 years, and one-third of them were 56 years or older years (Table 1). About three-quarters of the respondents were from rural areas. Males were more likely to be highly educated (12% vs. females 4%), currently employed (86% vs. females 11%) and currently married (97% vs. females 72%) than females, and these differences were statistically significant. Overweight/obesity prevalence was also higher among females (18% vs. males 9%). One-third of the respondents were from Dhaka—the central region. Television and mobile phone ownership were about 42 and 80%, in the study population (not shown).

**Table 1:** Characteristics* of study population and important sex differences (weighted estimates)

Characteristics	Male (*N* = 3870)	Female (*N* = 3955)	*p* value
Mean age (years)	51.9 (51.5–52.4)	50.9 (50.5–51.4)	0.37
Age category			
Younger (35–55 years)	65.7 (64.0–67.4)	69.1 (67.4–70.7)	<0.01
Older (56 years or more)	34.3 (32.5–36.0)	30.9 (29.3–32.6)	
Area of residence			
Rural	76.2 (74.8–77.6)	77.1 (75.7, 78.4)	0.17
Urban	23.8 (22.4–25.2)	22.9 (21.6, 24.3)	
Educational attainment			
No education or pre-school	37.8 (35.8–39.9)	58.6 (56.5–60.7)	<0.001
Primary school (1–5 years)	28.0 (26.3–29.8)	25.5 (23.8–27.2)	
Secondary or higher (≥6 years)	34.2 (32.1–36.4)	15.9 (14.3–17.7)	
Wealth status			
Poorest/poorer	39.4 (37.0–41.9)	37.9 (35.5–40.4)	0.175
Middle	19.5 (18.0–21.2)	20.1 (18.4–21.8)	
Richer/richest	41.1 (38.9–43.3)	42.0 (39.7–44.4)	
Working status			
Currently working	85.8 (84.5–87.1)	11.0 (9.9–12.4)	<0.001
Not working	14.2 (12.9–15.5)	89.0 (87.6–90.1)	
Marital status			
Currently married	97.1 (96.4–97.6)	71.5 (69.9–73.1)	<0.001
Others	2.9 (2.4–3.6)	28.5 (26.9–30.1)	
Nutritional status (BMI)			
Normal (18.5–24.9)	62.0 (60.2–63.8)	52.9 (51.1–54.7)	<0.001
Thin (<18.5)	28.8 (27.1–30.6)	29.4 (27.6–31.2)	
Overweight/obese (25.0/+)	9.2 (8.2–10.2)	17.7 (16.3–19.3)	
Region of residence			
Dhaka (central)	32.1 (30.5–33.6)	32.2 (30.6–33.8)	<0.01
Chittagong (south-east)	15.8 (14.7–16.9)	18.1 (17.0–19.2)	
Sylhet (east)	5.6 (5.1–6.1)	5.9 (5.4–6.4)	
Barisal (south)	5.9 (5.4–6.4)	6.0 (5.5–6.6)	
Khulna (west)	13.3 (12.4–14.2)	12.8 (11.9–13.7)	
Rajshahi (mid-west)	14.8 (13.6–16.1)	14.1 (13.2–15.1)	
Rangpur (north-west)	12.6 (11.8–13.4)	11.0 (10.2–11.8)	
Ever checked blood pressure (prior to the survey)	70.7 (68.7–72.7)	75.8 (73.8–77.6)	<0.001
Informed by GP/nurse about having high BP	10.8 (9.7–12.0)	21.3 (19.7–22.9)	<0.001
Antihypertensive medication use	7.1 (6.2–8.2)	14.3 (13.1–15.7)	<0.001

Except mean age, other estimates are prevalence with 95% confidence interval

About three-fourth of the study population had their BP measured prior to the survey. Table 1 also reveals frequency of BP measurement (prior to the survey) was higher among females (76%) than males (71%). Females were twice more likely to be aware about their own high BP (21% vs. males 11%). Overall, prescribed antihypertensive medicine use (self-reported) was also almost double among females compared with males (14% vs. 7% males).

**Fig. 1:**
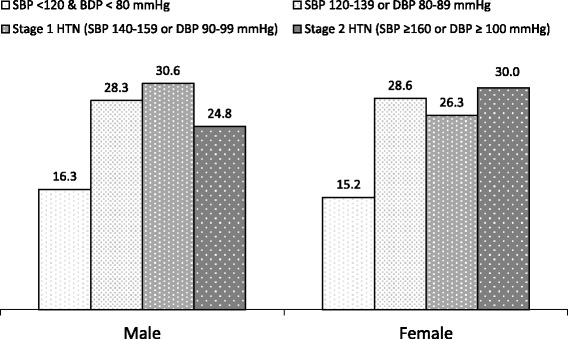
Blood pressure status (%) of adult male and female antihypertensive users in Bangladesh (weighted estimates). This descriptive figure shows that overall about half of the Bangladeshi male (45%) and female (44%) antihypertensive users had their BP controlled (SBP <140 and DBP <90 mmHg). About one-in-three of these self-reported antihypertensive users, male (25%) and female (30%), had stage 2 hypertension (SBP ≥160/DBP ≥100 mmHg) despite taking prescribed BP-lowering medications

Figure 1 shows 45% of male and 44% of female antihypertensive users had their BP controlled (SBP <140 & DBP <90 mmHg) and about one-in-three antihypertensive users had stage 2 hypertension (SBP ≥160/DBP ≥100 mmHg) despite taking prescribed medications.

Table 2 provides sex-specific information on prevalence (with 95% CI) of prior blood pressure measurement, hypertension awareness, antihypertensive use and control of blood pressure, by different factors. In general, older population, urban residents, individuals with higher socio-economic status and high BMI (overweight/obese) were more likely to check BP, aware about own high BP and use prescribed antihypertensive medications.

**Table 2:** Prevalence (with 95% CI) of prior BP measurement, own hypertension awareness, antihypertensive medication use and control of blood pressure (SBP <140 and DBP <90) among antihypertensive users, by different factors (weighted estimate)

	Ever measured blood pressure		Awareness about own high BP (from GP/nurse)		Antihypertensive use among aware hypertensives (self-reported)		BP control among antihypertensive users	
	Male	Female	Male	Female	Male	Female	Male	Female
	3869 (*N*)	3955 (*N*)	752 (*N*)	1260 (*N*)	417 (*N*)	840 (*N*)	269 (*N*)	562 (*N*)
Overall prevalence (%)	70.7 (68.7–72.7)	75.8 (73.8–77.6)	43.3 (39.5–47.3)	55.2 (51.7–58.5)	64.5 (58.2–70.2)	66.9 (62.6–70.8)	44.6 (37.9–51.6)	43.8 (39.1–48.6)
Age category								
Younger (35–55 years)	68.7 (66.2–71.1)	75.1 (72.8–77.2	36.1 (31.0–41.5)	54.9 (50.5–59.1)	56.5 (48.1–64.4)	66.3 (61.2–71.1)	43.1 (32.6–54.2)	49.7 (43.9–55.6)
Older (56 years or more)	74.6 (71.7–77.4)	77.4 (74.4–80.1)	51.1 (45.7–56.5)	55.5 (50.5–60.4)	71.4 (63.6–78.2)	67.6 (61.1–73.4)	45.7 (36.7–55.0)	35.7 (28.9–43.0)
Area of residence								
Rural	67.7 (65.2–70.1)	72.6 (70.2–74.9)	40.4 (35.9–45.1)	53.6 (49.4–57.7)	62.0 (53.9–69.5)	63.1 (57.7–68.2)	48.7 (39.9–57.5)	42.5 (36.9–48.3)
Urban	80.4 (77.0–83.5)	86.5 (83.3–89.2)	49.9 (42.9–56.9)	59.1 (53.1–64.8)	69.1 (59.1–77.5)	76.1 (70.3–81.2)	37.9 (27.7–49.3)	46.4 (38.2–54.9)
Educational attainment								
No education or pre-school	59.8 (56.5–62.9)	70.6 (68.0–73.1)	32.6 (26.2–39.8)	49.8 (45.3–54.4)	58.4 (N/A)	63.7 (57.8–69.2)	52.4 (39.1–65.4)	42.3 (35.6–49.1)
Primary school (1–5 years)	71.5 (68.4–74.4)	78.7 (75.5–81.6)	43.1 (35.2–51.3)	58.0 (51.9–63.8)	61.8 (50.7–71.8)	70.7 (63.1–77.3)	29.5 (19.4–42.1)	43.1 (34.9–51.8)
Secondary school or higher (≥6 years)	82.2 (79.7–84.5)	90.0 (86.9–92.5)	51.2 (44.8–57.6)	70.0 (63.2–76.0)	69.0 (60.8–76.1)	70.5 (62.5–77.4)	47.9 (38.2–57.7)	47.9 (38.5–57.4)
Wealth status								
Poorest/poorer	59.2 (55.9–62.4)	63.7 (60.5–66.9)	32.1 (N/A)	44.2	53.9 (N/A)	53.2 (N/A)	60.6 (N/A)	45.7 (N/A)
Middle	70.4 (66.1–74.4)	74.6 (70.7–78.3)	35.8 (N/A)	56.7 (48.6–64.5)	57.0 (N/A)	65.5 (55.8–74.1)	49.1 (N/A)	44.3 (N/A)
Richer/richest	82.0 (79.5–84.2)	87.2 (85.1–89.1)	51.7 (46.4–56.9)	61.3 (56.8–65.5)	70.1 (62.6–76.6)	74.1 (69.5–78.2)	39.3 (31.9–47.3)	43.0 (36.9–49.3)
Working status								
Currently working	69.3 (67.0–71.4)	66.9 (61.9–71.6)	41.5 (36.9–46.3)	53.7 (43.4–63.7)	60.4 (53.2–67.3)	71.6 (57.2–82.7)	46.7 (38.6–54.9)	50.3 (32.6–67.9)
Not working	79.6 (75.6–83.1)	76.9 (74.9–78.8)	48.6 (41.3–56.0)	55.3 (51.7–58.8)	75.8 (65.5–83.9)	66.5 (62.0–70.7)	40.1 (28.1–53.3)	43.2 (38.4–48.1)
Marital status								
Currently married	70.5 (68.4–72.5)	75.6 (73.3–77.7)	43.1 (39.2–47.1)	56.5 (52.5–60.5)	63.9 (57.6–69.8)	65.8 (61.1–70.3)	43.7 (36.8–50.9)	48.2 (42.7–53.7)
Others	79.3 (69.9–86.4)	76.4 (73.4–79.1)	48.1 (N/A)	52.9 (47.5–58.2)	77.8 (N/A)	68.8 (62.5–74.5)	62.3 (N/A)	35.8 (28.7–43.6)
Nutritional status (BMI)								
Normal (18.5–24.9)	72.4 (70.0–74.7)	76.8 (74.4–79.0)	42.7 (37.8–47.7)	52.9 (48.5–57.2)	64.1 (56.3–71.2)	65.2 (59.5–70.4)	37.6 (29.7–46.2)	51.1 (44.6–57.7)
Thin (<18.5)	61.6 (57.8–65.2)	65.5 (62.2–68.8)	32.9 (N/A)	45.2 (37.8–52.9)	56.5 (N/A)	53.5 (43.1–63.7)	51.5 (N/A)	27.6 (17.2–41.2)
Overweight/obese (≥25.0)	89.5 (85.5–92.5)	91.0 (88.5–93.1)	59.1 (49.3–68.1)	67.7 (61.8–73.1)	72.2 (60.5–81.6)	77.1 (71.6–81.8)	55.1 (41.5–68.0)	43.0 (N/A)
Region of residence								
Dhaka (central)	75.1 (70.9–78.8)	81.0 (76.9–84.4)	43.1 (35.3–51.4)	55.2 (47.7–62.5)	67.0 (52.7–78.7)	66.8 (58.0–74.6)	44.0 (30.2–58.8)	42.5 (32.2–53.4)
Chittagong (south-east)	66.4 (60.2–72.1)	72.4 (66.8–77.3)	57.0 (45.6–67.6)	66.2 (57.8–73.7)	62.6 (48.4–74.9)	64.3 (53.2–74.0)	51.3 (36.5–65.9)	57.9 (48.4–67.0)
Sylhet (east)	67.3 (60.4–73.5)	69.3 (63.2–74.8)	60.4 (46.4–72.9)	65.0 (53.6–74.9)	71.6 (53.4–84.7)	79.1 (65.4–88.3)	32.9 (22.0–45.9)	48.3 (37.3–59.6)
Barisal (south)	66.6 (60.2–72.4)	70.7 (65.2–75.6)	52.1 (38.6–65.2)	55.6 (46.5–64.3)	66.8 (51.3–79.4)	74.2 (63.1–82.8)	61.5 (43.0–77.2)	28.5 (21.1–37.3)
Khulna (west)	80.7 (76.2–84.5)	86.9 (83.3–89.8)	34.5 (26.9–43.0)	51.8 (44.5–59.0)	59.0 (43.4–73.0)	58.7 (48.9–67.8)	36.8 (21.4–55.6)	34.5 (25.0–45.4)
Rajshahi (mid-west)	63.5 (58.2–68.6)	68.8 (62.9–74.1)	45.0 (34.3–56.2)	59.6 (51.9–66.8)	63.3 (47.0–77.1)	74.1 (64.0–82.2)	45.9 (27.3–65.7)	48.5 (37.0–60.2)
Rangpur (north-west)	66.3 (61.0–71.2)	68.7 (63.4–73.6)	30.7 (23.1–39.6)	36.3 (28.5–45.0)	62.2 (45.8–76.3)	60.0 (46.8–71.9)	37.2 (19.7–58.8)	26.8 (15.3–42.5)

*N/A* stratum with single sampling unit

Table 2 illustrates that overall females with hypertension were more likely to be informed about having high BP by a GP or nurse, than that of male hypertensive (55% vs. males 43%, *p* < 0.001). When looking at self-reported use of prescribed antihypertensive medication by these ‘aware’ individuals, no statistical significance was found between females and males (67% females and 65% males 65%, *p* = 0.39).

Antihypertensive use was significantly higher among females in the urban areas compared with females living in rural areas (76% urban vs. rural 63%, *p* < 0.05). Non-working males were more likely to use antihypertensive medications compared with working males (76 vs. 60%, *p* 0.003). Between non-working males and non-working females, antihypertensive use was also significantly higher among males (males 76% vs. female 67%, *p* value 0.042). We knew only 11% of the females were working; nevertheless, antihypertensive medication use was higher among working females than working males (72% vs. males 60%, *p* = 0.094), though not statistically significant.

Table 2 further reveals more than half of the respondents did not have their blood pressure controlled despite taking antihypertensive medications. Females in their older age (≥56 years) were less likely to have their BP controlled than older males (36% vs. males 46%, *p*<0.05). No such statistically significant difference was observed between younger males and younger females.

Significant regional variances have also been observed for BP check, hypertension awareness and use of prescribed antihypertensive medications.

Table 3 presents AOR with 95% CI for factors affecting BP check-up, hypertension awareness, antihypertensive use and control of hypertension among antihypertensive users. The findings demonstrate interesting similarities and differences in the strength of associations for males and females.

**Table 3:** Adjusted odds ratio (with 95% CI) for factors affecting BP measurement, hypertension awareness, antihypertensive medication use and control of hypertension (weighted estimates)

	Ever measured blood pressure		Awareness about own high BP		Antihypertensive use among aware hypertensives		BP control among antihypertensive users	
Characteristics	Male	Female	Male	Female	Male	Female	Male	Female
Age category								
Younger (35–55 years)	1.0	1.0	1.0	1.0	1.0	1.0	1.0	1.0
Older (56 years or more)	1.38** (1.11–1.71)	1.32* (1.06–1.65)	2.62*** (1.73–3.97)	1.41* (1.01–1.98)	2.40** (1.46–3.94)	1.18 (0.77–1.81)	1.07 (0.55–2.08)	0.67 (0.42–1.05)
Area of residence								
Rural	1.0	1.0	1.0	1.0	1.0	1.0	1.0	1.0
Urban	1.21 (0.93–1.56)	1.35 (0.99–1.84)	0.93 (0.62–1.39)	0.88 (0.63–1.23)	1.13 (0.60–2.16)	1.45 (0.95–2.22)	0.58 (0.30–1.13)	1.24 (0.74–2.07)
Educational attainment								
No education or pre-school	1.0	1.0	1.0	1.0	1.0	1.0	1.0	1.0
Primary school (1–5 years)	1.49*** (1.23–1.81)	1.29* (1.02–1.62)	1.34 (0.76–2.34)	1.19 (0.84–1.68)	1.22 (0.58–2.55)	1.05 (0.70–1.59)	0.36* (0.14–0.90)	0.88 (0.53–1.46)
Secondary or higher (≥6 years)	2.20*** (1.77–2.75)	2.19*** (1.51–3.16)	1.71* (1.01–2.89)	1.96** (1.26–3.03)	1.36 (0.66–2.79)	0.73 (0.42–1.27)	1.15 (0.46–2.87)	1.21 (0.67–2.18)
Wealth status								
Poorest/poorer	1.0	1.0	1.0	1.0	1.0	1.0	1.0	1.0
Middle	1.39** (1.10–1.75)	1.36* (1.07–1.72)	1.06 (0.59–1.89)	1.37 (0.90–2.10)	1.48 (0.71–3.10)	1.72* (1.03–2.86)	0.73 (0.26–2.03)	0.79 (0.40–1.51)
Richer/richest	1.74*** (1.37–2.20)	2.26*** (1.76–2.91)	1.64 (0.96–2.82)	1.38 (0.99–1.93)	2.21* (1.10–4.44)	2.78*** (1.48–3.50)	0.41* (0.16–1.03)	0.56 (0.30–1.06)
Working status								
Currently working	1.0	1.0	1.0	1.0	1.0	1.0	1.0	1.0
Not working	1.52** (1.11–2.07)	1.86*** (1.42–2.42)	0.99 (0.61–1.62)	0.95 (0.59–1.53)	1.74 (0.90–3.35)	0.75 (0.37–1.51)	0.50* (0.25–1.01)	0.84 (0.43–1.61)
Marital status								
Currently married	1.0	1.0	1.0	1.0	1.0	1.0	1.0	1.0
Others	1.39 (0.82–2.34)	1.22 (0.99–1.51)	1.05 (0.41–2.67)	0.86 (0.62–1.19)	1.53 (0.34–6.87)	1.14 (0.77–1.69)	1.61 (0.36–7.14)	0.83 (0.53–1.29)
Nutritional status (BMI)								
Normal (18.5–24.9)	1.0	1.0	1.0	1.0	1.0	1.0	1.0	1.0
Thin (<18.5)	0.71*** (0.59–0.84)	0.69*** (0.58–0.83)	0.62 (0.37–1.04)	0.79 (0.55–1.13)	0.72 (0.37–1.41)	0.65 (0.40–1.08)	1.44 (0.67–3.12)	0.34** (0.17–0.68)
Overweight/obese (25.0/+)	2.23*** (1.50–3.31)	2.10*** (1.56–2.83)	1.81** (1.18–2.78)	1.54** (1.14–2.10)	1.35 (0.75–2.45)	1.61* (1.09–2.39)	2.74** (1.28–5.84)	0.62 (0.35–1.08)
Region of residence								
Dhaka (central)	1.0	1.0	1.0	1.0	1.0	1.0	1.0	1.0
Chittagong (south-east)	0.61** (0.43–0.87)	0.58** (0.40–0.83)	1.37 (0.74–2.54)	1.54 (0.95–2.51)	0.67 (0.24–1.63)	0.78 (0.43–1.43)	1.33 (0.55–3.18)	2.45** (1.30–4.64)
Sylhet (east)	0.75 (0.52–1.09)	0.60** (0.41–0.87)	1.93 (0.96–3.88)	1.61 (0.91–2.86)	1.33 (0.47–3.74)	1.87 (0.84–4.19)	0.54 (0.25–1.15)	1.72 (0.88–3.33)
Barisal (south)	0.67* (0.47–0.97)	0.62* (0.43–0.89)	1.47 (0.70–3.10)	1.16 (0.70–1.91)	1.03 (0.39–2.71)	1.77 (0.90–3.47)	2.00 (0.68–5.90)	0.53* (0.28–0.99)
Khulna (west)	1.36 (0.95–1.96)	1.61* (1.09–2.37)	0.66 (0.39–1.11)	0.87 (0.57–1.32)	0.76 (0.31–1.87)	0.64 (0.37–1.11)	0.54 (0.20–1.45)	0.57 (0.29–1.11)
Rajshahi (mid-west)	0.61** (0.44–0.86)	0.58** (0.41–0.84)	0.94 (0.54–1.63)	1.27 (0.80–2.01)	0.87 (0.34–2.22)	1.62 (0.83–3.16)	0.69 (0.26–1.84)	1.37 (0.70–2.68)
Rangpur (north-west)	0.82 (0.58–1.15)	0.70* (0.49–1.00)	0.66 (0.37–1.18)	0.56* (0.34–0.91)	0.98 (0.38–2.51)	0.84 (0.42–1.68)	0.52 (0.18–1.45)	0.47* (0.22–1.02)

*p* value ***<0.001; **0.001–<0.01; *0.01−0.05

For example, while both older males and females were more likely to have their blood pressure checked or aware about own hypertension, compared to younger groups, no statistically significant difference was observed between younger and older females in antihypertensive medications use. On the other hand, antihypertensive use was 2.4 times higher among older males, compared with younger males. We have also found older female antihypertensive users were 37% less likely to have their BP controlled (AOR 0.67, 95% CI 0.42–1.05) compared with younger females, even though the difference was not statistically significant.

We found no statistically significant differences in BP measurement, hypertension awareness or antihypertensive medication use between urban and rural dwellings in adjusted analysis, both for males and females. Adjusted analysis further shows female antihypertensive users in the urban areas had 24% higher odds (AOR 1.24, 0.74–2.02) of having their BP controlled whereas urban males had 42% lower odds (AOR 0.56, 0.30–1.13) of having their BP controlled compared with their rural peers, even though the associations were statistically non-significant.

Higher educational attainment was associated with significantly higher odds of having BP check-up and hypertension awareness. However, higher educational attainment did not show any significant association with better antihypertensive medication practice. Primary schooling (1–5 years) was associated with poor BP control (statistically significant for males only), but post primary education showed statistically non-significant better BP control (15% for males and 21% for females), compared with individuals with no education.

Overall, increased wealth status was associated with better checking of BP, hypertension awareness and use of antihypertensive mediation—but poor BP control. Males and females using antihypertensive mediation in the upper two wealth quintiles (richer/richest) had 59% (AOR 0.41, 0.16–1.03) and 44% (AOR 0.56, 0.30–1.06) lower odds of having their BP controlled, compared with the poorest/poorer individuals.

Table 3 also reveals that non-working males had 1.74 times higher odds of using antihypertensive medication compared with currently working males, but non-working females had 25% less odds of using antihypertensive (AOR 0.75, 0.37–1.51), compared with currently working females, even though the observed associations were not statistically significant. BP control is poor among both non-working males and females but statistically significant for non-working males only (AOR 0.50, 0.25–1.01).

Overweight/obesity was associated with more BP check-ups, hypertension awareness, and antihypertensive medication use, for both males and females. Overweight/obese males were 2.74 times more likely to have their BP controlled (AOR 2.74, 1.28–5.84) compared with normal BMI males. On the other hand, compared with normal BMI females, both thin (AOR 0.34, 0.17–0.68) and overweight/obese (AOR 0.62, 0.35–1.08) females had poor BP control.

Residents of Khulna region in the west were most likely to have their BP checked. BP check-up was significantly low in other regions, compared with Dhaka—the central region. Khulna and Rangpur regions lagged behind in hypertension awareness. Antihypertensive medication use was low in Chittagong, Khulna and Rangpur regions—compared with Dhaka—even though statistically not significant. Blood pressure control varied between the regions; however, for both males and females, the poorest blood pressure control was observed in Khulna and Rangpur regions.

## Discussion

To our knowledge, this is the first report in Bangladesh that has explored the gap between males and females in terms of high BP awareness, antihypertensive medications use and control of BP. Our analysis showed, while checking BP and hypertension awareness were better among women compared with men, the advantage was not translated into better antihypertensive medication practice among women. Furthermore, females not involved in any income generation or in the poorest/poorer wealth quintiles were less likely than their male peers to use antihypertensive medication. Our findings highlight a gender inequality in treatment affordability for expensive antihypertensive medications.

In general, we found higher hypertension awareness and medication use among women, similar to a previous report from Bangladesh. However, our findings differed from Rahman et al. [23] who reported hypertensive women were twice more likely (AOR 2.72) than men to get treatment. Our sex-specific analysis revealed that antihypertensive medications use was almost similar (65–67%) between hypertensive males and females, who were aware about their condition. For those in need, awareness is the precursor of getting treatment. Therefore, simply looking at medication use data, which was about double among women, would not identify the unmet need in the community. We have also showed non-working women were disadvantaged because of their low use of antihypertensive medication, compared with non-working males.

Our findings substantiated the general consensus that while women are knowledgeable about having hypertension, this does not translate into adequate BP control [24]. Rahman et al. reported better hypertension control (AOR 2.22) among Bangladeshi female antihypertensive users [23]. Contrary to their findings, our sex-disaggregated analysis showed older females had poor BP control compared with older males (36 vs. 46%) and younger females (AOR 0.67). It should be noted that our analysis agreed well with the BDHS 2011, which reported about 56% of the antihypertensive medication users did not achieve BP control [21], whereas much lower BP control rate (males 28%, females 34%) was reported by Rahman et al. We also found overweight males had better BP control while poor BP control was observed among overweight females. Our findings reiterate the importance of sex-disaggregated analysis and reporting.

Higher prevalence of blood pressure measurement, hypertension awareness and antihypertensive use were observed in urban areas. In the adjusted analysis, we also found urban living was associated with higher odds of having BP check or using antihypertensive medications than rural living, for both males and females even though the associations were not statistically significant. Studies in low-income countries reported better antihypertensive treatment and hypertension control in urban areas [7]. A systematic review and meta-analysis in India also reported significantly better hypertension awareness (42 vs. 25%), treatment (38 vs. 25%) and control (20 vs. 11%) in urban areas compared with rural area [25]. In our national representative sample, only about one-third of the respondents were from urban areas. Greater urban–rural mix-up due to urban migrations may have also contributed to our findings.

Few studies have reported strong association between education and hypertension treatment in low-income countries [26]. We found increased hypertension awareness with higher education. However, the observed positive effect of higher education on higher antihypertensive medication use and better hypertension control were mostly statistically non-significant. It should be kept in mind that prevalence of secondary or higher education was very low, one-in-ten among males and about 4% among females.

We observed poor BP control among the wealthier group, irrespective of sex. Higher hypertension and diabetes prevalence in the upper two wealth quintiles have also been reported in Bangladesh [27]. Wealthier people are more likely to be overweight/obese and engaged in sedentary lifestyle and less physical labour. However, NCD burden among lower income individuals might be a significant challenge in future due to observed lower consumption of fruits and vegetables, low hypertension awareness and low treatment compliance in Bangladesh [28].

Bangladesh has achieved dramatic success, especially over the last 20 years, in closing gender inequalities in female mortality and malnutrition. A number of community-based interventions from the government and Non-Government Organisations (NGOs) including outreach sites and satellite clinics, community health workers, female education programmes and women empowerment through micro-credit schemes could be attributed for such achievements [29]. During 1993–2011, coverage of antenatal care at public health facilities, which includes routine BP measurement, doubled in Bangladesh [30]. However, despite an increase in social standing and the increased accessibility of health system, Bangladeshi women still lack empowerment and financial freedom and gender inequity is prevalent [31].

The high prevalence of undiagnosed hypertension in Bangladesh highlights the critical need for increased awareness and education about NCDs and their risk factors at the community. Non-pharmacologic interventions that bring about effective behaviour change for hypertension control include dietary sodium reductions, weight loss and physical activity [32]. Every 10 mmHg decrease of SBP has been associated with about one-fifth reduction of major CVD events [33]. Policy makers need to roll-out preventive strategies, especially targeting high-risk populations like disadvantaged and older females, to mitigate the growing burden of hypertension.

Increased hypertension awareness does not guarantee life style modifications or antihypertensive treatment. Poor adherence to pharmacological and non-pharmacological therapy is one of the commonest causes of uncontrolled hypertension [34]. BDHS 2011 did not collect detailed information on antihypertensive medication, duration of use or compliance—which limit our ability to further explore high uncontrolled hypertension prevalence in the community. A previous report from Bangladesh showed non-adherence to antihypertensive treatment was very high, around 85% [35]. At present, the essential services package (ESP) of the government of Bangladesh does not include antihypertensive medications [36]. Therefore, Government should take steps for developing simplified antihypertensive guidelines for the country and train primary care service providers on appropriate management of hypertension in the community.

Bangladesh government has recently rolled out hypertension screening and counselling through the newly established community clinics [30]. Low-cost, reliable, automated BP measurement devices, like WHO recommended Omron HEM-SOLAR [37], could be used to increase hypertension detection and management. Innovative public health approaches like engaging non-physician health workers (task-shifting), electronic decision support system (smartphone), BP monitoring and behaviour change (mobile health counselling) could be evaluated targeting high-risk population in the community.

Higher risk of having uncontrolled hypertension in some particular regional areas require further exploration from a public health perspective. Khulna region, situated in the coastal belt, is affected by high arsenic concentration [38], high salinity [39] and poverty [23]. Rangpur, situated in the northern part, along the belt of Jamuna river, has been affected by chronic iodine deficiency [40]. Programme personnel and policy makers should also explore the coverage and accessibility of public health services in the some areas.

Our study has several important strength and limitations. The main strength of our study is a large nationally representative sample of the adult population. However, despite the robust methodology, we need to be aware of the cross-sectional nature of the study which limits our ability to infer causal relationships. Blood pressure and blood glucose concentration often coexist, and these conditions are interlinked [41]. But due to cross-sectional nature of data, we did not consider diabetes in risk factor analysis.

## Conclusions

Our sex-disaggregated analysis revealed blood pressure check-up and hypertension awareness were better among Bangladeshi women, but the advantage was not translated into better antihypertensive medication practice, compared to men. We have also found gender inequality in treatment affordability for expensive antihypertensive medications, especially for females not involved in any income generation or in the poorest/poorer wealth quintiles. We have emphasized the importance of sex-disaggregated analysis and reporting. We believe such findings would help in raising hypertension awareness and designing appropriate gender-specific public health interventions in Bangladesh and other developing countries (Table 4).

**Table 4:** Summary table

What is known about the topic	What this study adds
• Low prevalence of treatment and high prevalence of uncontrolled hypertension in Bangladesh • Women who are hypertensive were more likely to receive treatment and more likely to have their BP controlled. • Residents in western or north-western part of Bangladesh were more likely to have uncontrolled hypertension	• Sex-disaggregated analysis and reporting are important in recognizing existing inequity and unmet needs in hypertension management • Higher blood pressure check-up and hypertension awareness among Bangladeshi women did not translate into better antihypertensive medication practice compared to men. • Inequality in treatment affordability for expensive antihypertensive medications, especially for females not involved in any income generation or in the poorest/poorer wealth quintiles. • Bangladesh and other developing countries should design appropriate gender-specific public health intervention to promote hypertension awareness and treatment • Poor BP control in western and north-west geographical regions requires further scientific exploration

## Abbreviations

ANC: Antenatal care
AOR: Adjusted odds ratio
BDHS: Bangladesh Demographic and Health Survey
BMI: Body mass index
CI: Confidence interval
CKD: Chronic kidney disease
CVD: Cardiovascular disease
DBP: Diastolic blood pressure
ESP: Essential services package
GP: General practitioner
MDG: Millennium Development Goal
mmHg: Millimetre of mercury
NCD: Non-communicable disease
NGO: Non-Government Organisations
SBP: Systolic blood pressure
WHO: World Health Organization
